# Ambulatory monitoring of blood pressure and pregnancy outcome in pregnant women with white coat hypertension in the third trimester of pregnancy

**Published:** 2013-01-01

**Authors:** Nahid Shahbazian, Heshmatollah Shahbazian, Razieh Mohammadjafari, Mahsan Mousavi

**Affiliations:** ^1^Fertility Infertility and Perinatology Research Center, Ahvaz Jundishapur University of Medical Sciences, Ahvaz, Iran; ^2^Department of Nephrology, Ahvaz Jundishapur University of Medical Sciences, Ahvaz, Iran; ^3^Department of obstetric & gynecologist, Ahvaz Jundishapur University of Medical Sciences, Ahvaz, Iran

**Keywords:** Hypertension, White coat hypertension, Holter monitoring

## Abstract

**Introduction:** If the blood pressure of a pregnant woman is ≥140/90 mmHg at the clinic, but her ambulatory blood pressure is less <135/85 mmHg at daytime and <125/75 at night and her average ambulatory in 24 hours is <130/80 mmHg, her high blood pressure at clinic is considered white coat hypertension.

**Objectives:** To evaluate the value of ambulatory blood pressure monitoring in pregnant women.

**Patients and Methods:** This prospective cohort study was conducted in Imam-Khomeini hospital of Ahwaz, Iran between 2011 to 2012. A total of 105 pregnant women who had blood pressure of higher than 140/90 mmHg during the third trimester of pregnancy were monitored. Thirty five women with white coat hypertension, 35 women with gestational hypertension and 35 women with normal blood pressure were followed. The data were analyzed using the Kolmogorov-Smirnov test, Pearson correlation coefficient and Chi-square tests.

**Results:** The prevalence of white coat hypertension was 31.3%. The maternal and neonatal outcomes and laboratory examinations in white coat hypertension were similar to the normal blood pressure, but the frequency of caesarean section was more than the other two groups.

**Conclusion:** The findings of the study indicate the efficacy of 24 hour holter monitoring of blood pressure and using it more comprehensively , compared to the limited visits.

**Implication for health policy/practice/research/medical education:**

Prospective cohort study indicated the efficacy of 24-hour holter monitoring of blood pressure and using it in a more comprehensive way compared to the limited visits.

## Introduction

Hypertension (HTN) occurs in approximately 5-10% of all pregnancies. Among a wide variety of the causes of high blood pressure (BP) in pregnancy, preeclampsia syndrome, whether alone or in the form of added on the chronic hypertension, is considered as the most hazardous condition (1).

In developed countries, hypertensive disorders are responsible for around 16% of maternal deaths, and the important concern is that more than half of these deaths are associated with high BP that can be prevented (2-4). Gestational HTN is defined when BP of ≥140/90 mmHg is detected for the first time during pregnancy (5).

All current clinical criteria are usually based on the mean of at least two seated BP measurements during two outpatient visits. Generally, the results of home monitoring or 24-hour ambulatory BP measurements are lower than the clinical measurements (6-8). Since the mobile devices measure BP several times during the day and at night, these devices are considered as more comprehensive assessment tools compared with limited visits (9-14).

A growing body of evidence suggests that, the home monitoring, during work hours at office or ambulatory BP measurements have a better relationship with the end-organ damages compared to the measurements of the physicians’ office or clinics (15-17).

If the BP of a pregnant woman is ≥140/90 mmHg, but the ambulatory measured value during the day is lower than 135/85 mmHg, less than 125/75 mmHg at night, and during the first 24-hour is less than 130/80 mmHg, this BP is considered as white coat or office only HTN. White coat HTN happens due to the adrenergic transient response to the stressful condition of measuring the BP in the physician’s office (18-20).

One of the key variables that often remains far from the mind is the used method of the study and the necessity to standardize the measurements. White coat or office only HTN occurs in about 30% of the patients (21-26). Household tools or 24-hour ambulatory monitoring devices are often proper tools for those patients that have normal BP out of the physician’s office or clinic (15,27-29).

In the study conducted by Bellomo *et al*, it was found that, the prevalence of white coat hypertension was 29.2% (20). In another study conducted by Mc Grath *et al*, the outpatient monitoring of blood pressure in predicting the pregnancy outcome was evaluated. Adverse pregnancy outcomes, including the incidence of preeclampsia, intrauterine growth retardation (IUGR) and preterm delivery were compared between two groups of with white coat hypertension and those with gestational hypertension. Undesirable consequences were significantly more common in the gestational hypertension group (30).

## Objectives

The present study aimed to evaluate 24-hour ambulatory monitoring of blood pressure by holter monitoring devices in patients with white coat hypertension and also to evaluate the prevalence of white coat hypertension among the pregnant women and its effect on pregnancy outcomes.

## Patients and Methods

### Patients

This was a prospective cohort study. A total of 105 pregnant women, who had BP of more than 140/90 mmHg during the prenatal visits in the third trimester of pregnancy in two visits and at least two times with a time interval of five minutes, were studied.

The total number of samples was 105 women: thirty five pregnant women with white coat hypertension, 35 pregnant women with gestational hypertension and 35 pregnant women with normal blood pressure.

### Measurement of blood pressure

After explaining the objectives and details of the study, if the patients agreed to take part, the consent forms were obtained and they were enrolled. The BP measuring method included measuring twice with a five minute interval while the patient seated upright in a chair and t her arm kep at heart level. An adult size of BP cuff was used to measure blood pressure. The patient’s right arm was used to measure BP. Then these patients underwent the 24-hour ambulatory monitoring of blood pressure with a Holter monitoring device (Agillis, Australia). Holter monitoring device was comprised of a small and special pressure gauge, which at first was programmed and set by a computer and then it was connected to the patient. In the specified intervals while the patient was busy with everyday activities, every half hour during the day and every two hours during the night for 24 hours, the device was set to measure the blood pressure.

Out of the 105 pregnant women, who were undergoing Holter monitoring device, 47 people had negative holter results and 103 people had positive Holter results. Out of the 47 people with negative Holter results, 35 women who had blood pressure less than 125/75, 135/85 and 130/80 mmHg during the day, night, and 24 hours, respectively, entered the study as group of white coat hypertension (negative Holter group). Also, out of 103 patients with positive results during the outpatient monitoring, 35 patients, who had blood pressure higher than the above mentioned values, entered the study as gestational hypertension (positive Holter group). Furthermore, 35 pregnant women who were diagnosed with normal blood pressure alone, entered the study as the control group.

### Laboratory tests

To compare the outcomes of pregnancy, up to the end of the pregnancy, these three groups were followed every two weeks and their blood pressure, complete blood count (CBC), liver enzymes, creatinine and proteinuria were assessed.

Finally, duration of pregnancy, the rout of delivery (cesarean section, normal vaginal delivery with or without induction), the incidence of preeclampsia and eclampsia, the preterm labor, duration of maternal and neonatal hospitalization, the weight of the their babies, intrauterine fetal death and laboratory examinations among three groups were studied and compared among the studied groups.

### Definition of preeclampsia and eclampsia

Preeclampsia was defined as blood pressure of equal to or more than 140/90 mmHg after 20 weeks of gestation along with proteinuria equal to or more than 300 mg in 24 hours. Eclampsia was defined as having a seizure episode non-attributable to other causes in women with preeclampsia. Preterm labor was defined as the baby’s birth before 37 weeks of the pregnancy; intrauterine fetal death was defined as fetal death after 20 weeks of gestation; and low birth weight (LBW) was defined as birth weight between 500 and 2500 g.

### Ethical issues

The research followed the tenets of the Declaration of Helsinki. Written informed consent was obtained from all patients. This study was approved by ethical committee of Ahvaz Jundishapur University of Medical Science.

### Statistical analysis

The Kolmogorov-Smirnov test was used to identify the normal distribution of variables and ANOVA test was used to compare the means of the three groups. Furthermore, Pearson correlation coefficient and Chi-square tests were used for comparing qualitative variables between the three groups. Data analysis was performed using SPSS version 19.0 software and the P-values ≤ 0.05 were considered statistically significant.

## Results

In our study the prevalence of white coat hypertension was 31.3%. Among the variables investigated, the time of termination of pregnancy, the baby’s weight at birth, the number of babies and maternal hospitalization days (Table 1). The mean weight of babies, born in the gestational hypertension group was significantly less than the other two groups (p= 0.004). The neonatal hospitalization duration (Table 1) in the gestational hypertension group was significantly more than the other two groups (p= 0.01). As shown in Table 2, the Cesarean section procedure in the white coat hypertension group was significantly more common than the other two groups. There was no significant difference in the average proportion of the incidence of intrauterine death (Table 3; p= 0.13) among the groups. The average incidence of preeclampsia and eclampsia (Table 3) in the gestational hypertension group were significantly more than the other two groups (p= 0.01, p= 0.0003, p= 0.012, and p= 0.005, respectively). Normal vaginal delivery was significantly more common in the normal pregnancy group. Induction procedure was significantly more common in the gestational hypertension group (p= 0.012).

**Table 1:** Mean ± SD duration of gestation, birth weight and infant and maternal hospital stay in the pregnant women studied

**Group**	**Duration of gestation (Week and day)**		**Birth weight (Gram)**		**Length of infant 's hospital stay (Day)**		**Length of mother 's hospital stay (Day)**	
	**Mean**	**SD**	**Mean**	**SD**	**Mean**	**SD**	**Mean**	**SD**
White coat hypertension	39w+4d	0.382	3300	1.987	1.7	1.481	1.5	1.912
Gestational hypertension	3d‏+38w	1.281	2900	1.754	4.2	1.658	5.9	2.156
Normal blood pressure	39w+3d	0.3101	3350	1.645	1.2	2.893	1.2	2.893
P-value		0.01		0.004		0.01		0.0003

**Table 2:** Comparison of the rout of delivery in pregnant women

**Group**	**Cesarean (%)**	**Normal vaginal delivery**	**Inductive**	**P-value**
White coat HTN	45.7	48.6	5.7	0.012
Gestational HTN	40	48.6	11.4	
Normal blood pressure	11.4	82.9	5.7	

**Table 3:** Comparison of the incidence of pre eclampsia, eclampsia, preterm labor, low birth weight, intrauterine fetal death, reduced platelet, increased Keratin abnormal liver function test, urinary aluminum and preterm delivery in pregnant women studied

**Group**	**Pre-eclampsia (%)**	**Eclampsia (%)**	**Preterm labor**	**Low birth weight**	**Intrauterine fetal death**	**Reduced platelet**	**Increased Keratin**	**Abnormal liver test**	**Urinary aluminum**
White coat HTN	5.7	0	0	0	0	0	0	0	0
Gestational HTN	60	2.9	14.3	14.3	5.7	14.3	25.7	60	57.1
Normal blood pressure	8.5	0	0	0	0	0	0	0	0
P-value	0.0001	0.0001	0.005	0.005	0.13	0.005	0.0001	0.0001	0.0001

The average ratio of occurrence of reducing the number of platelets, increased creatinine, the incidence of abnormal liver function test and albumin in the random urine (Table 3) in the gestational hypertension group was significantly higher than the other two groups (p= 0.005, p= 0.0001, p= 0.0001, and p= 0.0001, respectively).

## Discussion

This study showed that, the prevalence of white coat hypertension in pregnant women was 31.3%. That mentioned prevalence was slightly more than non-pregnant population (20). Only in the study conducted by Parati *et al*., prevalence of white coat hypertension was more common than our study (24). However, the frequency of caesarean of pregnants with white coat hypertension was more often than pregnants with normal blood pressure and the those with high blood pressure of pregnancy. The cause of an increase in the caesarean section in the group of white coat hypertension was hard to interpret, although in the group of white coat hypertension may be due to the decision on how to end a pregnancy based on the measurement of blood pressure in the physician’s office or clinic. Another cause for it, is the common and a normal blood pressure increase in passing of blood pressure around the pregnancy term.

In the study conducted by Bellomo *et al*. on how to end a pregnancy in the white coat hypertension, it was shown that the frequency of the termination of the pregnancy using the cesarean section technique in the group of the white coat hypertension was more than the gestational hypertension and normal blood pressure groups (20). In the study by Mc Grath *et al*. the consequences of prenatal in white coat hypertension was desirable, and undesirable outcomes such as low birth weight, IUGR and premature pregnancy in the gestational hypertension group were more common (30). In Parati *et al*. study of pregnancy outcomes in white coat hypertension, adverse outcomes such as prematurity and preterm labor and intrauterine fetal death (IUFD) in white coat hypertension were not observed (24).

In our study, the abnormal laboratory results, including reducing the number of platelets, increased creatinine, the abnormal liver function tests and albumin in the random urine of pregnant women with gestational hypertension were observed, however, the impaired laboratory results were not observed in people with white coat hypertension and the pregnant women with normal blood pressure. Similar results were also obtained in other studies, including the study of Bellomo *et al.* (20). Also abnormal results were not observed in the laboratory examinations of white coat hypertension in the study conducted by Hodgkinson *et al.* (7).

The impaired liver enzymes, platelet count, and creatinine were not observed in the laboratory examinations in the study of Mark *et al*. on the white coat hypertension (15). Our study showed that, risks of preeclampsia and eclampsia were higher in pregnant women with gestational HTN than pregnant women with white coat hypertension or pregnant women with normal blood pressure. Additionally, the duration of the pregnancy and duration of the mother’s hospitalization and preterm labor was more common in the gestational hypertension group than other groups. In the study of Bellomo *et al.* on maternal results of the white coat hypertension, the risks of preeclampsia and eclampsia in white coat hypertension group were significantly less than gestational hypertension group (20). Jose *et al.* studied the adverse maternal outcomes such as preeclampsia and eclampsia in white coat hypertension and observed that they were significantly lower in gestational hypertension group (11).

In analyzing the neonatal results in our study, low birth weight and the hospitalization duration of newborns in the hospital in the gestational hypertension group was more than the other two groups. In this study, we have shown that the weight of the baby has a direct relationship with the results of the monitoring Holter set. Therefore, in the positive Holter set group, the baby weight was less than the negative Holter set group. Bellomo *et al,* also considered the incidence of low birth weight and the lower Apgar of the minute one and more duration of hospitalization of baby in hospital in the white coat hypertension group. They found that, these were significantly less in the gestational hypertension group (20). In the study of Mark *et al.*, adverse neonatal outcomes such as low weight at birth and the lesser duration of hospitalization in white coat hypertension group, were significantly lower than the gestational hypertension (15).

## Conclusion

Our study showed the higher incidence of white coat hypertension and therefore the utility of ambulatory 24-hour blood pressure monitoring in its detection. In white coat hypertension, pregnancy outcome was similar to the pregnant women with normal blood pressure. The 24-hour ambulatory blood pressure monitoring will provide more comprehensive information and it will followed by ensuring the favorable outcome of pregnancy in women with white coat hypertension.

## Limitations of the study

One of the limitations of the study was the limited number of visits of patients. Principally, it was due to the need for more visits to select the patients (for example, three visits instead of two visits on arrival to study). It was also necessary to educate pregnant women and encourage them to come for more follow up visits during pregnancy.

## Authors’ contributions

Main draft write up and editing by MM. Important intellectual content and critical revision by NS, RM and HS.

## Conflict of interests

The authors declared no competing interests.

## Ethical consideration

Ethical issues (including plagiarism, misconduct, data fabrication, informed consent, double publication) have been completely observed by the authors.

## Funding/Support

This paper has been derived from the residential thesis of this study was granted by Ahvaz Jundishapur university of medical sciences and department of Gynecology and Obstetrics, fertility and perinatology research center, Ahvaz Jundishapur university of medical sciences and research consultation center.

## Acknowledgements

Hereby we would sincerely appreciate the Holter monitoring Center, Imam Khomeini Hospital, and patients participating in this study and all the ones who helped us in this study. The authors also, appreciate and thank the Research Deputy vice-chancellor for research affairs of the Ahvaz Jundishapur University of Medical Sciences, particularly the Research Consultation Center (RCC) for technical support.
